# A caregiver-reported global severity assessment in pediatric atopic eczema: the Comano score

**DOI:** 10.1186/s13052-020-0805-9

**Published:** 2020-04-23

**Authors:** Mattia Giovannini, Davide Geat, Gabriele Barlocco, Riccardo Pertile, Francesca Mori, Cesare Filippeschi, Elio Novembre, Mario Cristofolini, Ermanno Baldo

**Affiliations:** 1 Allergy Unit, Department of Pediatrics, Anna Meyer Children’s University Hospital, Florence, Italy; 2 Post-Graduate School of Dermatology, Section of Dermatology and Venerology, Department of Medicine, University of Verona, Verona, Italy; 3 “Giovan Battista Mattei” Research Institute, Stenico, Italy; 4 Department of Clinical and Evaluative Epidemiology, Trento Health Service, Trento, Italy; 5 Dermatology Unit, Department of Pediatrics, Anna Meyer Children’s University Hospital, Florence, Italy

**Keywords:** Scoring system, Atopic dermatitis, Comano score, Children, Person-centered care

## Abstract

Atopic eczema (AE) is the most common inflammatory skin disease in infancy and its prevalence is rising worldwide. It has a wide social impact on the affected children and their families’ lives. AE can have a chronic and heterogeneous course, with periods of remission and relapse of the clinical manifestations. For this reason, its severity assessment through standardized outcome measures becomes a fundamental guide for health professionals, who can manage AE following evidence-based medicine principles in their everyday clinical practice or in clinical trials.

Several scoring systems have been recognized to assess the clinical manifestations of AE, both from the physician’s and the patient’s point of view. Despite the scoring systems standardized for adults, there are very few published options about the expression of a patient/caregiver-centered global severity assessment specifically for pediatric AE. For this reason, the aim of our study was to evaluate a new, quick, user-friendly and feasible caregiver-reported global severity assessment for pediatric AE. Based on a 0–10 numerical rating scale in pediatric AE, we named this scoring system the Comano score.

We carried out a cross-sectional observational study enrolling a total of 867 patients aged from 1 to 16 years (males 49.5%, mean patient’s age 5.9 years, standard deviation ±3.6 years) with a previous doctor-confirmed diagnosis of AE, who underwent balneotherapy at Comano Thermal Center (Comano, Trentino, Italy). A strong correlation between Comano score and SCORing Atopic Dermatitis (SCORAD) was observed (*r* = 0.74, *p* < 0.0001).

According to our results, the Comano score may be a promising new tool for the expression of a caregiver-reported global severity assessment in pediatric AE. However, further data are needed to confirm our preliminary findings before health professionals can use this scoring system in their everyday clinical practice to manage pediatric AE. Still, as a patient-focused measure, the Comano score may facilitate delivering person-centered care so as to define a measure for a clinical impact that can be meaningful to the subject, which is gaining importance in modern medicine.

To the Editor,

Atopic eczema (AE) is the most common inflammatory skin disease of infancy, affecting up to 20% of the pediatric population in Western countries, with its prevalence rising worldwide [1]. It has a wide social impact on the affected children and their families’ lives, as it jeopardizes their daily activities, their sleep and their mental health, in addition to its direct/indirect economic costs. Moreover, the impact of AE is significantly increased by comorbidities, especially asthma [2].

AE is characterized by a chronic and heterogenous course, with periods of remission and relapse [1]. Therefore, AE severity assessment through standardized outcome measures is a fundamental guide for health professionals, who can manage AE following evidence-based medicine principles in their everyday clinical practice or in clinical trials.

According to the systematic review carried out by the international multidisciplinary initiative Harmonising Outcome Measures in Eczema (HOME), among 16 eligible tools to assess the clinical signs of AE, the Eczema Area and Severity Index (EASI) and objective SCORing Atopic Dermatitis (oSCORAD) were the ones with the highest validation. Thus, they should be the choice to evaluate outcome measures for AE signs in clinical trials [3–5].

Patient-focused measures are getting more and more important in modern medicine, characterized by a person-centered care in order to define a measure for a clinical impact that can be meaningful to the subject. On this note, a cross-sectional survey-based study analyzed different AE patient-reported global severity assessment measures in adults [6]. As a result, numeric rating scale (NRS) itch, Patient-Oriented SCORing Atopic Dermatitis (PO-SCORAD) and Patient-Oriented Eczema Measure (POEM) were proven to be reliable scoring systems to evaluate AE severity in clinical practice [7, 8]. Despite the validity these tools showed, they are also quite time-consuming and not so easy for patients to understand. This can negatively impact their feasibility and use in real-life practice. However, a new, quick, easy and manageable tool called Patient-Reported Global Severity of Atopic Dermatitis has also been recently validated for clinical use in adult patients [9].

Despite the scoring systems validated for adults, there are very few published options about the expression of a patient/caregiver-centered global severity assessment specifically for AE in children. For this reason, the aim of our study was to evaluate a new, quick, user-friendly and feasible caregiver-reported global severity evaluation for pediatric AE. Based on a 0–10 NRS, we named this tool the Comano score.

We carried out a cross-sectional observational study, enrolling pediatric patients aged 1 to 16 years, with a previous doctor-confirmed diagnosis of AE, who underwent balneotherapy at Comano Thermal Center (Comano, Trentino, Italy) from April to October 2014. All the patients with medical conditions that may contraindicate or negatively influence balneotherapy, such as cutaneous diseases (e.g. bacterial, viral or fungal infections, ulcers or skin cancer) or systemic pathologies (e.g. infectious diseases, malignancies, cardiac diseases and immunodeficiencies) were excluded from the balneotherapy, as required by the center policy and by the principles of good clinical practice in thermal medicine, and they were therefore excluded also from the study. An informed, written consent was obtained from all parents/guardians prior to including the patients in the study.

The caregivers assessed the AE clinical manifestations severity through a 0–10 NRS, by answering the following physician’s question: “How would you describe your AE clinical manifestations severity from 0 to 10, where 0 (minimum score) means absence of AE signs and symptoms and 10 (maximum score) means maximum severity of AE signs and symptoms?”. This score is composed of 10 units with the minimal expressible difference of 1 unit.

After that, either a dermatologist or a pediatrician evaluated the patients’ clinical history and carried out the necessary dermatological examination to assess the AE clinical manifestations severity through SCORing Atopic Dermatitis (SCORAD) [5]. This scoring system evaluates AE using three different criteria: extension of the involved skin surface expressed as percentage (objective item), intensity (objective item; through the assessment of each of the following elements on a 0–3 balance: erythema, dryness, edema/papulation, oozing/crusting, excoriation, lichenification) and subjective symptoms (pruritus during the day and sleep loss, each on a 0–10 scale). The scores are then merged together according to the formula: “SCORAD (minimum score 0 – maximum score 103; score composed of 103 units with the minimal expressible difference of 1 unit) = extent (0-100)/5 + intensity (0-18) x 3.5 + subjective items (0-20)” [5].

Data on the assessment of the AE clinical manifestations severity were collected using 4 categories (0–3, 4–6, 7–8, 9–10) for the Comano score and 5 categories (< 15, 15–30, 31–40, 41–60, > 60) for SCORAD. The Comano score concurrent validity compared with SCORAD was assessed through simple linear regression by evaluating the Pearson’s correlation coefficient between them. In the data reporting phase, descriptive statistics continue variables were presented as counts, mean and standard deviation (SD) and categorical variables as counts and percentages. The correlation was classified according to the Pearson’s coefficient: 0.10–0.29 was considered as a small correlation, 0.30–0.49 a medium correlation and 0.50–1.0 a large correlation. Statistical data analysis was carried out using SAS software (SAS institute, Cary, North Carolina, United States of America).

A total of 867 patients were included in the study, 49.5% males and with a mean patient’s age of 5.9 years (SD ± 3.6 years). Comano score and SCORAD of the study population are shown in Table 1. A strong correlation between the Comano score and SCORAD was observed (r = 0.74, *p* < 0.0001) (Fig. 1).

**Table 1:** SCORAD and Comano score of the study population

Patients	***n*** (%)
	867 (100%)
**SCORAD**	
0–15	357 (41.2%)
15–30	237 (27.3%)
30–40	141 (16.3%)
40–60	91 (10.5%)
> 60	41 (4.7%)
**Comano score**	
0–3	369 (42.6%)
4–6	300 (34.6%)
7–8	167 (19.3%)
9–10	31 (3.6%)

*SCORAD*: SCOring Atopic Dermatitis

**Fig. 1:**
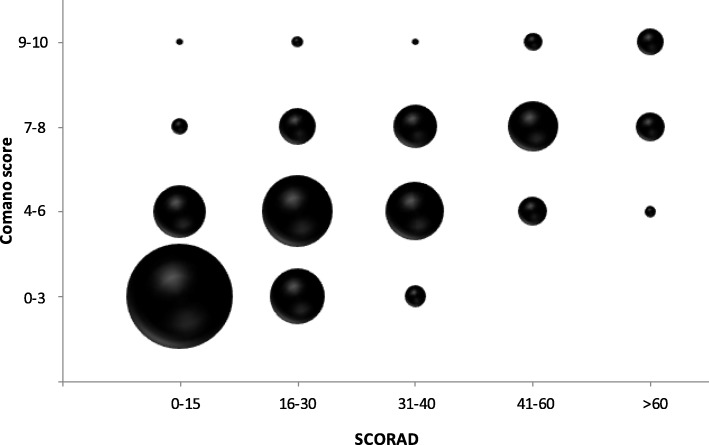
Correlation between SCOring Atopic Dermatitis (SCORAD) and Comano score

Despite the great epidemiological impact of AE in pediatric patients, there are only a few reported articles regarding patient/caregiver-centered global severity assessment in pediatric age.

A longitudinal descriptive single-center study published by Lee JY et al. [10] demonstrated Atopic Dermatitis Symptom Score (ADSS), included in a smartphone application, as a useful tool for patients or their parents to assess the severity of AE in children on a daily basis. ADSS focuses on 6 different parameters: 4 signs (dryness, erythema, oozing, oedema) and 2 symptoms (sleep disturbance and itching). They are measured on a scale from 0 (“mild”) to 4 (“very severe”) with a potential score ranging from 0 to 24 units with a minimal expressible difference of 1 unit. The authors pointed out how the score had strong intra-observer (test-retest) reliability [intraclass correlation coefficient = 0.82; 95% confidence interval (95% CI): 0.70–0.90]. ADSS also demonstrated a statistically significant concurrent validity (*r* = 0.64, *p* < 0.0001) and responsiveness (*r* = 0.56, *p* < 0.0001) compared to SCORAD.

A prospective observational multicenter experience reported by Koh MJ et al. [11] studied the correlation between Patient Eczema Severity Time (PEST), carried out daily by a caregiver, and SCORAD, in the evaluation of AE severity in pediatric patients from 6 months to 6 years of age. PEST is a picture-based score with 5 images associated with caregiver/patient-reported global severity assessment ranging from 1 (“not at all unhappy”) to 5 (“extremely unhappy”). The authors highlighted a weak-moderate correlation between this score and SCORAD in children (ranging from *r* = 0.22 at baseline to *r* = 0.58 at week 12). However, they pointed out the advantages of using PEST to describe the changes in the AE severity over time, considering its higher responsiveness to modifications compared to SCORAD (PEST 33.3%; 95% CI: 26.0–40.5% versus SCORAD 13.8%; 95% CI: 9.5–18.1% of scale). However, it is worth noting that PEST has not been studied in children over 6 years of age.

The communication of a patient-centered global severity assessment for AE is challenging in pediatric patients, as parents/guardians are the ones in charge of the patients’ care. Therefore, caregiver-centered severity evaluation seems to be the optimal alternative. From our results, the Comano score may be a promising new tool to express a caregiver-reported global severity assessment in pediatric AE. It is based on a simple 0–10 NRS, which may become a quick, easy and feasible scoring system for pediatric patient’s parents/guardians.

The high number of participants is a strong point of our study, as it enabled us to demonstrate the significant concurrent validity of the Comano score compared to SCORAD in a heterogeneous study population, with patients suffering from a wide range of AE severity. However, our study inevitably has several limitations, too, mainly driven by the clinical setting in which it was implemented, i.e. admission visits for balneotherapy. For this reason, we did not have the opportunity to follow up the patients. On the contrary, it would have been helpful to study the dynamic changes of Comano score and SCORAD over time, in accordance to AE signs and symptoms modifications, and then compare the Comano score’s responsiveness to SCORAD. Moreover, data about the intraobserver (test-retest) reliability would have been necessary as well to appropriately validate it. Finally, receiver-operating characteristic curve analyses should be carried out to assess the Comano score cut-off point associated with severe AE (SCORAD ≥40).

Therefore, further data are needed to confirm our preliminary findings before health professionals can use this promising scoring system in their everyday clinical practice to manage pediatric AE. Nevertheless, as a patient-focused measure, the Comano score may facilitate delivering person-centered care so as to define a measure for a clinical impact that can be meaningful to the subject, which is gaining importance in modern medicine.

## Abbreviations

95% CI: 95% Confidence Interval
ADSS: Atopic Dermatitis Symptom Score
AE: Atopic Eczema
EASI: Eczema Area and Severity Index
HOME: Harmonising Outcome Measures in Eczema
NRS: Numerical Rating Scale
oSCORAD: objective SCORing Atopic Dermatitis
PEST: Patient Eczema Severity Time
POEM: Patient-Oriented Eczema Measure
PO-SCORAD: Patient-Oriented SCORing Atopic Dermatitis
SCORAD: SCORing Atopic Dermatitis
SD: Standard Deviation

## References

[1] Odhiambo JA, Williams HC, Robertson CF, Asher MI, Clayton TO (2009). Global variations in prevalence of eczema symptoms in children from ISAAC phase three. J Allergy Clin Immunol.

[2] Zinelli C, Caffarelli C, Strid J, Jaffe A, Atherton DJ (2009). Measurement of nitric oxide and 8-isoprostane in exhaled breath of children with atopic eczema. Clin Exp Dermatol.

[3] Schmitt J, Langan S, Deckert S, Svensson A, von Kobyletzki L (2013). Assessment of clinical signs of atopic dermatitis: a systematic review and recommendation. J Allergy Clin Immunol.

[4] Leshem YA, Hajar T, Hanifin JM, Simpson EL (2015). What the eczema area and severity index score tells us about the severity of atopic dermatitis: an interpretability study. Br J Dermatol.

[5] Kunz B, Oranje AP, Labrèze L, Stalder JF, Ring J (1997). Clinical validation and guidelines for the SCORAD index: consensus report of the European task force on atopic dermatitis. Dermatology.

[6] Silverberg JI, Margolis DJ, Boguniewicz M, Fonacier L, Grayson MH, et al. Validation of five patient-reported outcomes for atopic dermatitis severity in adults. Br J Dermatol. 2019. 10.1111/bjd.18002. 10.1111/bjd.18002 30972740

[7] Stalder JF, Barbarot S, Wollenberg A, Holm EA, De Raeve L (2011). Patient-oriented SCORAD (PO-SCORAD): a new self-assessment scale in atopic dermatitis validated in Europe. Allergy.

[8] Charman CR, Venn AJ, Ravenscroft JC, Williams HC (2013). Translating patient-oriented eczema measure (POEM) scores into clinical practice by suggesting severity strata derived using anchor-based methods. Br J Dermatol.

[9] Vakharia PP, Chopra R, Sacotte R, Patel N, Immaneni S (2018). Validation of patient-reported global severity of atopic dermatitis in adults. Allergy.

[10] Lee JY, Kim M, Yang HK, Kim HM, Cho J (2018). Reliability and validity of the atopic dermatitis symptom score (ADSS). Pediatr Allergy Immunol.

[11] Koh MJ, Giam YC, Liew HM, Foong AY, Chong JH (2017). Comparison of the simple patient-centric atopic dermatitis scoring system PEST with SCORAD in young children using a Ceramide dominant therapeutic moisturizer. Dermatol Ther (Heidelb).

